# Controlling dispersion forces between small particles with artificially created random light fields

**DOI:** 10.1038/ncomms8460

**Published:** 2015-06-22

**Authors:** Georges Brügger, Luis S. Froufe-Pérez, Frank Scheffold, Juan José Sáenz

**Affiliations:** 1 Department of Physics, University of Fribourg, Chemin du Musée 3, Fribourg CH-1700, Switzerland; 2 Depto. de Física de la Materia Condensada, Instituto Nicolás Cabrera and Condensed Matter Physics Center (IFIMAC), Universidad Autónoma de Madrid, Fco. Tomas y Valiente 7, Madrid 28049, Spain; 3 Donostia International Physics Center (DIPC), Paseo Manuel Lardizabal 4, Donostia-San Sebastian 20018, Spain

## Abstract

Appropriate combinations of laser beams can be used to trap and manipulate small particles with optical tweezers as well as to induce significant optical binding forces between particles. These interaction forces are usually strongly anisotropic depending on the interference landscape of the external fields. This is in contrast with the familiar isotropic, translationally invariant, van der Waals and, in general, Casimir–Lifshitz interactions between neutral bodies arising from random electromagnetic waves generated by equilibrium quantum and thermal fluctuations. Here we show, both theoretically and experimentally, that dispersion forces between small colloidal particles can also be induced and controlled using artificially created fluctuating light fields. Using optical tweezers as a gauge, we present experimental evidence for the predicted isotropic attractive interactions between dielectric microspheres induced by laser-generated, random light fields. These light-induced interactions open a path towards the control of translationally invariant interactions with tuneable strength and range in colloidal systems.

## Abstract (web summary)

Natural dispersion forces acting between molecules and particles arise from electromagnetic fields generated by quantum and thermal fluctuations. Here, Brügger *et al.* show that isotropic dispersion forces between colloidal particles can be induced, controlled and tuned with artificial, fluctuating laser light fields.

## Graphical abstract

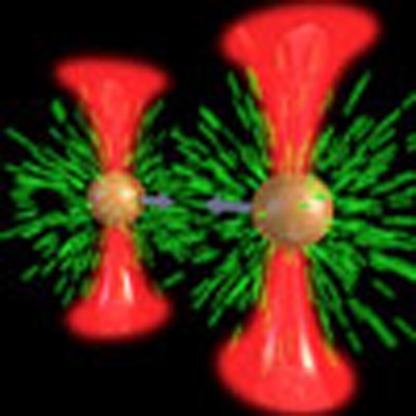

The familiar isotropic dispersion forces between neutral objects arise from random electromagnetic waves generated by equilibrium quantum and thermal fluctuations^1^,^2^,^3^,^4^. Depending on the context, these forces are known as non-retarded van der Waals–London, Casimir–Lifhsitz and, more generally, Casimir forces^1^,^2^,^3^,^4^. The interplay between Casimir forces and electrical double-layer forces, which forms the basis of the famous Derjaguin-Landau-Verwey-Overbeek (DLVO) theory^1^ describing the forces between charged surfaces in a liquid medium, plays a key role in the colloidal behaviour observed in biological fluids (for example, proteins, biopolymers and blood cells), foodstuffs (for example, dairy, thickeners, emulsions and creams) or suspensions (for example, pharmaceuticals, slurries, paints and inks)^5^,^6^. Colloids have also been shown to be extremely well suited for the study of phenomena such as crystallization, the glass transition, fractal aggregation and solid–liquid coexistence^7^,^8^,^9^. External control of isotropic interactions in colloidal systems is therefore of key importance. Temperature-sensitive swelling of microgel particles offers control over soft repulsive forces, but the process is slow, shows hysteresis^10^ and the properties of the colloids are altered while swelling. In some cases, magnetic and dielectric dipolar forces can be induced by external fields but these interactions are strongly anisotropic, leading to the formation of chains or anisotropic domains^11^. Other ways to control colloidal interactions usually involve the change of composition: by adding and removing electrolytes, the range of electrostatic repulsions can be tuned, and by dissolving macromolecules of appropriate size, attractive depletion forces can be induced^8^,^9^. However, despite their widespread and successful use, these strategies are still tedious and slow, and do not provide the level of control over interaction forces that, as discussed here, could be achieved by using external laser fields.

Intense light fields can be used to trap and manipulate small particles^12^,^13^,^14^ as well as to induce significant optical binding (OB) forces^13^,^15^,^16^, which, in general, are not translationally invariant, showing a strong anisotropy that depends on the interference landscape of the external fields^16^. Here we show that artificially generated random fields with appropriate spectral distribution can provide control over attractive and repulsive isotropic (and translationally invariant) interactions with tuneable strength and range. In contrast with Casimir interactions, where the forces are dominated by the material's response at low frequencies, our results open a new way to explore the peculiar optical dispersion of small particles and artificial metamaterials by selecting the spectral range of the random field. As an example, we predict that the interactions between semiconductor particles with relatively high refractive index can be tuned from attractive to strongly repulsive when the external frequency is tuned near the first magnetic Mie resonance^17^.

Using optical tweezers as a gauge, we present experimental evidence for the predicted isotropic attractive interactions between dielectric microspheres induced by laser-generated, quasi-monochromatic random light fields. We note that isotropic optical forces between particles act instantly and can therefore also be applied dynamically. This can potentially be useful to anneal defects in periodic structures such as photonic crystals, to increase the effective temperature by optically shaking particles or to stabilize non-equillibrium phases such as supercooled liquids and, in general, to control the self-assembly and phase behaviour of colloidal particle assemblies on nano- and mesoscopic length scales^6^,^7^.

## Results

### General theory of random-light-induced interactions

Early work by Boyer^18^ derived Casimir interactions between small polarizable particles from classical electrodynamics with a homogeneous and isotropic classical random electromagnetic field having the spectral density of quantum blackbody radiation including the zero-point radiation field. Here we extend these ideas to external artificial random fields with arbitrary spectral density, obtaining an explicit expression for the interactions between two arbitrary dielectric objects, which allows a compact description of random-light-field-induced interaction forces from dipolar (atomic or nanometre scale) to macroscopic objects. As a limiting case, when the spectral density of the random field corresponds to that of quantum blackbody radiation, we recover the exact trace formulae for Casimir interactions between arbitrary compact objects^19^,^20^. Related trace expressions have also been obtained to describe non-equilibrium Casimir interactions between objects held at different temperatures^21^,^22^.

We first analyse the connection between random-light-induced interaction forces and Casimir interactions between two arbitrary objects. We assume that object/particle A is at the origin of coordinates and particle B is displaced at a distance *r* along the positive *z* axis in an otherwise transparent and non-dispersive homogeneous medium with real refractive index 
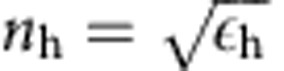
. We consider that the particles are illuminated by a quasi-monochromatic random field of frequency *ω*, which can be described as a superposition of plane waves with random phases and polarizations, propagating in all directions. Each particle can be seen as made of discretized *N*_A_ and *N*_B_, identical cubic elements of volume *v* and relative permittivity 
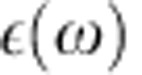
, which act as small polarizable units with an induced dipole proportional to the polarizing field, that is, 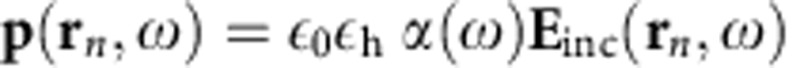, where *α*(*ω*) is the polarizability given by 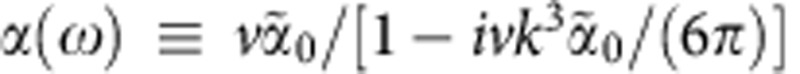 with 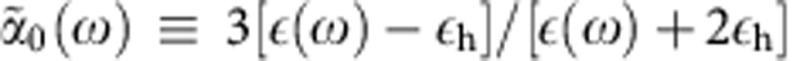. In the presence of a fluctuating polarizing field, ***E***_inc_(**r**,*t*), the induced dipoles are fluctuating quantities and the time-averaged force on particle B along the *z* axis may be written as^18^,^23^


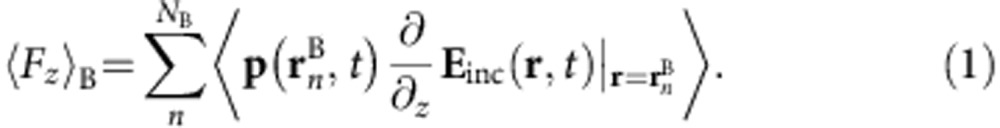


The total force can be seen as the sum of different contributions. Although for random illumination there is no net force on an isolated particle, the scattered field by B can be reflected back by particle A, leading to a series of multiple scattering events that give rise to a net interaction force between them. An additional contribution arises from the correlations between the induced dipoles. At a first sight, one could think that the incoming exciting fields on the two objects will be completely uncorrelated. However, in a random, statistically stationary and homogeneous electromagnetic field, the fields at two distant points are correlated, with a cross-spectral density of the correlations identical to that of blackbody radiation^24^ (see Supplementary Note 1). In the absence of absorption (when the relative permittivity 
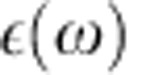
 and 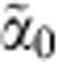 are real numbers), the sum of the two contributions lead to a conservative interaction force, which can be expressed in terms of the **T** matrix^25^ of each individual object (see Supplementary Notes 2 and 3)


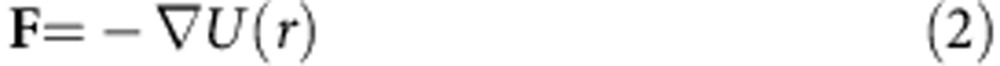



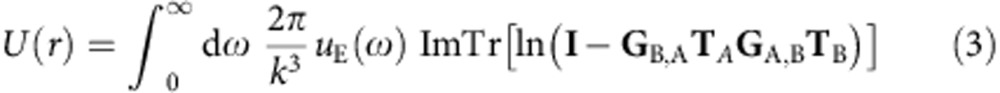


where [*u*_E_(*ω*)d*ω*]=*U*_E_(*ω*) is the energy of the fluctuating electric field per unit of volume and *k*=*n*_h_*ω*/*c* is the wave number (*c* is the speed of light in vacuum). 
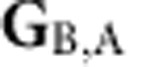
 is the Green tensor connecting the two objects and ‘Tr' stands for the trace of the matrix. The dependence of the interaction on distance is completely contained in 
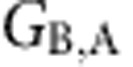
, whereas all the shape and material dependence is contained in the **T** matrices. The connection with Casimir interactions can be made through Boyer's approach: When the objects are in equilibrium with a quantum black body radiation, the energy density *U*_E_(*ω*) corresponds to the electric quantum zero-point fluctuations (at zero temperature and positive *ω*) given by^26^,^27^ *U*_E_(*ω*)=*u*_E_(*ω*)d*ω*=*ℏk*^3^/(4*π*^2^)*dω*. For absorbing (emitting) particles, we must include an additional contribution to the total force coming from the fluctuating dipoles and the corresponding radiated fields^28^ (linked through the fluctuation-dissipation theorem). Interestingly, in equilibrium, this additional contribution conspires with the force due to the field fluctuations, to give a total interaction potential, which is exactly given by equation (3), now including light absorption and emission (that is, 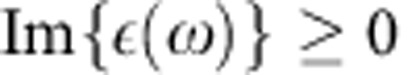), and we recover the exact Casimir interaction between arbitrary compact objects^19^,^20^. In contrast with the traditional Casimir forces, equation (3) opens the path towards complete control and tunability of isotropic dispersion forces between compact bodies by tailoring the spectral density of artificially generated random fields.

### Interactions between dipolar electric and magnetic particles

In the limit of small dipolar particles, equation (3) leads to Renne's result^29^ obtained from quantum-electrodynamic calculations, which is identical to Boyer's result^18^ based on classical electrodynamics (the more familiar Casimir–Polder result is recovered in the weak scattering limit). Recent theoretical works on OB between dipolar particles under non-coherent random illumination^30^,^31^ and on dipolar particles near fluctuating light sources^32^,^33^ suggested striking similarities between dipolar optical forces in random fields and Casimir interactions. However, from a practical point of view, the creation of isotropic random light fields and the direct detection of the resulting weak OB forces between particles at room temperature is challenging. For small non-absorbing particles, illuminated by a quasi-monochromatic random field, equation (3) takes the simple form


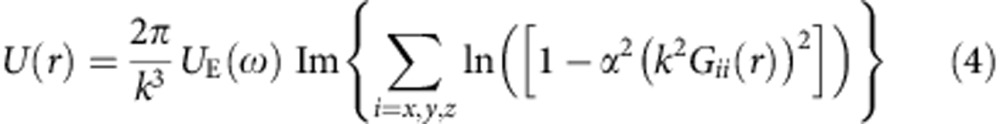


which can be shown to be equivalent to equation (11) in ref. 31 in the absence of absorption. In the weak scattering limit


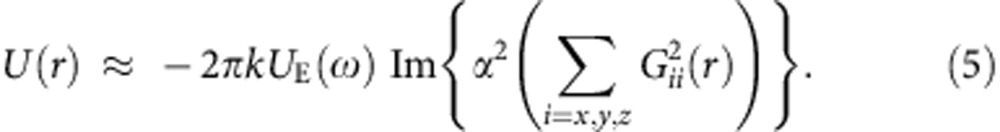


**Figure 1:**
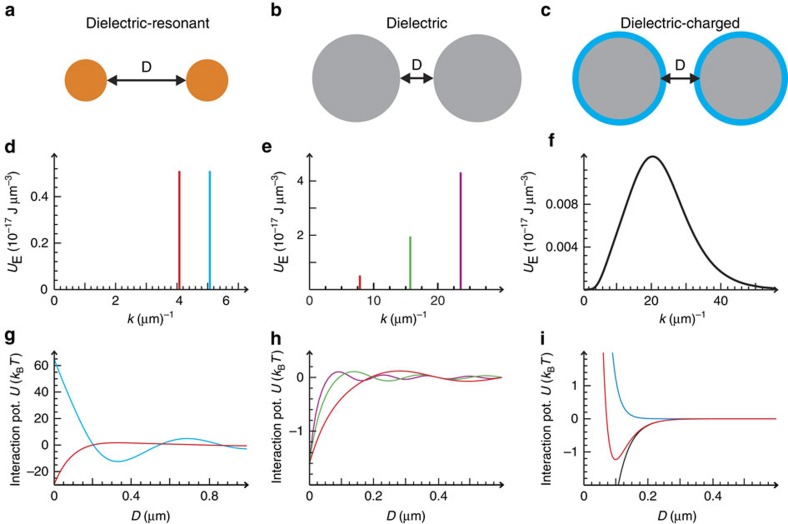
Tuneable interactions induced by random light fields. (**a**–**c**) Three representative examples of designer random-light-induced pair potentials *U*(*D*). (**d**–**f**) Electric field energy densities *U*_E_(*ω*) versus *k*=*n*_h_*ω*/*c*. Their corresponding interaction potentials are depicted in the third row (**g**–**i**). (**g**) Exact calculation based on equation (3) for non-absorbing semiconductor particles where the optical response at wavelengths 1.2–2 μm is well described by the electric and magnetic polarizabilities. Random light illumination allows for switching between strongly attractive (red) and strongly repulsive (blue) interactions. The examples show the interaction between two *R*=230 nm spheres (*n*=3.5) in water (*n*_h_=1.33). Based on equation (6), we calculate in **h** the interaction potential due to a monochromatic random light illumination between uncharged dielectric particles at different wavelengths (compare the corresponding colours). By changing the wavelength (or wave number *k*), the range of the interaction potential can be tuned precisely. (**i**) Properly designing the energy density spectrum of random light fields allows for exponentially attractive dispersion potentials (black curve). This property can be used to produce Morse-type potentials on a colloidal level using dielectric-charged particles. The exponential electrostatic double-layer repulsion calculated for a contact potential of 180 *k*_B_*T* and a Debye length of 20 nm (blue), and the exponentially attractive dispersion interaction (black) superimpose to a colloidal Morse potential (red).

The interaction energy far from resonance is attractive but always much smaller than *k*_B_*T* for realistic power densities, whereas at resonance the polarizability is purely imaginary (at resonance, 
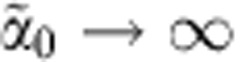
, that is, *α*^2^<0), which leads to an effective repulsion. The latter was shown to play a key role in understanding the collective behaviour of optical-trapped neutral atoms^34^. Plasmonic or polaritonic nanoparticles show a high real and imaginary polarizability close to a resonance, but this can lead to a significant increase of the temperature^35^. Non-absorbing semiconductor nanoparticles, with relatively high refractive index, or colloidal dielectric micron-sized particles would offer an attractive laboratory to verify our predictions. Semiconductor particles, for example, silicon (Si) spheres with index of refraction ∼3.5 and radius ∼200 nm^36^, present strong electric and magnetic dipolar resonances in telecom and near-infrared frequencies (that is, at wavelengths 1.2–2 μm) without spectral overlap with quadrupolar and higher-order resonances^17^. Assuming that the scattering by these Si particles can be described by just dipolar electric and magnetic fields, it is possible to obtain an exact closed expression for the interaction potential, equation (3), in terms of the electric and magnetic polarizabilities (see Supplementary Notes 4 and 5). Figure 1d,g illustrates how a monochromatic random illumination induces a pair potential between two identical 230-nm-radius Si nanospheres, which can be tuned from attractive off-resonance (*λ*∼2 μm) to strongly repulsive when the external wavelength is tuned near the first Mie's magnetic resonance (*λ*∼1.6 μm).

### Interactions between micron-sized dielectric particles

To derive a simplified theoretical expression for the interaction potential between micron-sized dielectric particles, we approximate equation (3) at lowest order in perturbation theory (see Supplementary Note 6). In this limit, the interaction energy can be seen as given by a pairwise interaction *u*(|**r**_B_−**r**_A_|) summed over the volume of the spheres, that is, similar to Hamaker's integral^1^,


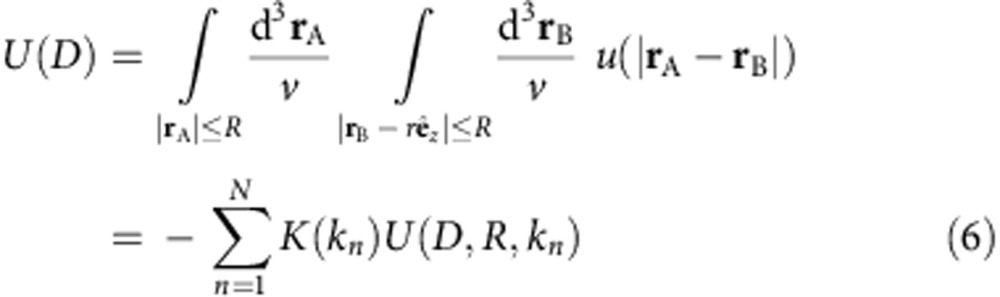


with size independent coefficients 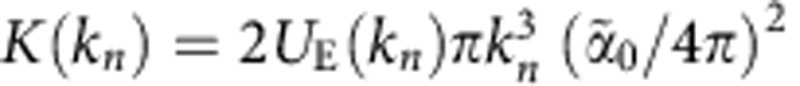 as a measure of the materials contribution to the interaction potential for a given spectral component *k*_*n*_=*n*_h_*ω*_*n*_/*c* of the random light field. *D*=*r*−2*R* denotes the gap distance between the surfaces and *r* is the distance between the centres of the two spheres. *U*_E_(*k*_*n*_) is the power density of the random light field for a specific wave number *k*_*n*_. For monochromatic illumination 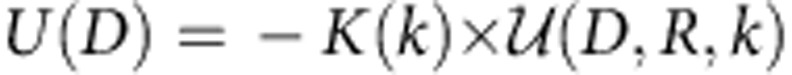 and in the limit of *kR*<<1<<*kD*, we recover the expected results for dipolar particles^15^,^31^ where the interaction energy is proportional to the squared volume of the particles. Interestingly, for relatively large particle sizes (in an intermediate regime 1≪*kD*≪*kR*) we find:


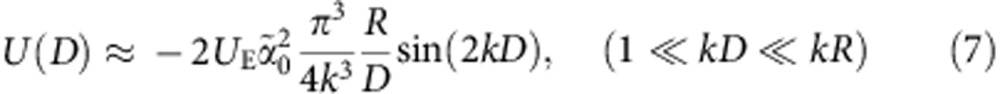


that is, for relatively large particle sizes, our approach predicts that the interaction energy should scale with the particle's radius. Otherwise equation (6) can be easily computed numerically. Figure 1h,i illustrates the predicted theoretical sensitivity of the induced pair interactions between low-index (*n*=1.68) micron-sized spheres to changes in the wavelength of the illuminating random field. As forces can be strongly frequency dependent, actual dispersion forces could be manipulated by tuning the spectrum of the random light field. As an example, in Fig. 1e,h we show that it is possible to tailor the range of an effective attractive dispersion interaction potential with constant depth by selecting different wavelengths and field-power densities (calculations are performed using equation (6)). Selecting an appropriate continuous spectrum of the random light fields it is also possible to design a nearly exponential attractive potential. Taking into account the well-known exponential electrostatic double-layer repulsion, it would theoretically be possible to induce an effective Morse-type interaction potential on a colloidal level, as shown in Fig. 1f,i.

### Experimental observation of random light interactions

**Figure 2:**
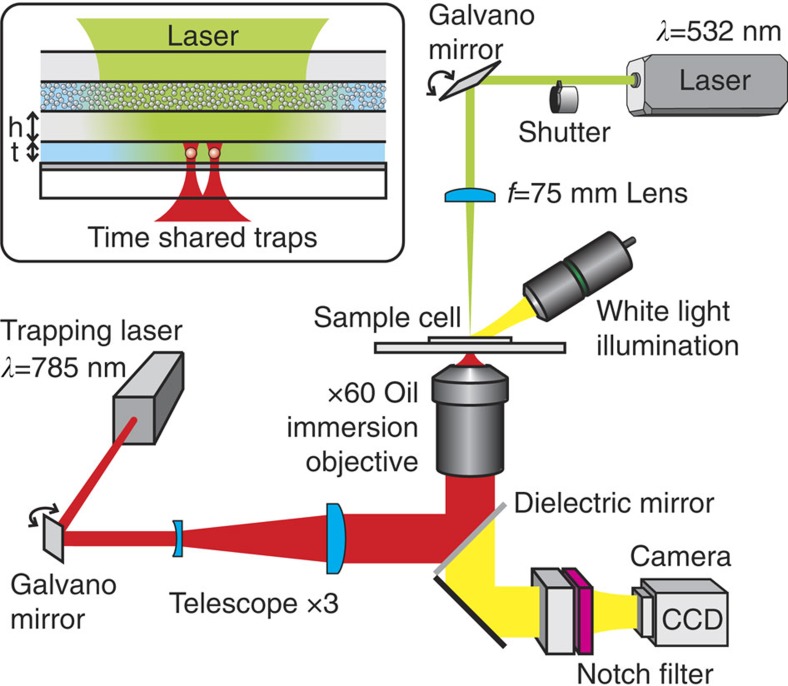
Experimental setup. An intense green laser (*λ*=532 nm) is weakly focused on one side of the sample cell using an *f*=75 mm lens. A turbid scattering layer of thickness 20 μm at the entrance of the cell creates a random field distribution inside a light-filled cavity of dimensions ∼(50 μm)^3^. From the opposite side, the sample cell is illuminated with a tightly focused near-infrared laser (*λ*=785 nm) creating a set of two time-shared optical traps. Both laser beams can be steered by galvano mirrors. A white light source is employed for the broadband illumination of the cell, allowing the tracking of the particles by video microscopy. Different filters are used to spectrally isolate the different optical paths. The interaction potential between the two trapped spheres is obtained by monitoring the thermal Brownian motion inside the optical traps with a digital camera (CCD). The inset shows an enlarged view of the sample cell: Two micron-sized colloidal particles suspended in water are trapped in the centre of a water-filled layer of thickness *t*=15 μm. The water layer and the turbid layer are separated by a glass wall of thickness *h*≃20 μm. The dichroic mirror at the bottom of the clear layer reflects >99% of the incoming light leading to multiple reflections inside the cavity.

Experimentally, the manipulation of relatively large, micron-sized dielectric particles requires high-intensity laser fields: in conventional optical tweezing experiments, the focused laser intensity *I*_0_=*P*/*A* required to tightly trap a micron-sized polystyrene sphere (refractive index *n*∼1.6) is on the order of mW μm^−2^ and the corresponding power density is *U*_E_=*n*_h_/*c*·*I*_0_. Therefore, to create random light fields with a comparable power density, the surface area *A* cannot exceed some tens of micrometres squared, for an incident laser power *P* on the scale of Watts. To this end, we have designed a miniaturized sample cell that allows for the simultaneous creation of a random light field in a small cavity by illumination with a strong green laser *λ*=532 nm and the observation of OB forces between two isolated microspheres (Fig. 2). We use the technique of time-shared optical tweezers^37^,^38^,^39^ in combination with umbrella sampling^40^, to probe the particle pair interaction potential *U*(*r*) of two melamine microspheres (*n*=1.68) as a function of their centre-to-centre separation distance *r*. The principle of this method is to trap, at the same time, two particles in two identical optical tweezers that are separated by a distance *r*_0_ using a near-infrared laser beam with a wavelength of 785 nm. By monitoring the relative motion of the trapped particles with a digital camera, information about the pair potential *U*(*r*) around *r*_0_ is gained. In contrast to vacuum, the interactions between particles suspended in water commonly involve both van der Waals and screened electrostatic repulsive interactions (Derjaguin-Landau-Verwey-Overbeek -DLVO- interactions). The latter ensures the stability against particle coagulation. Equally, in our measurements, this repulsive part dominates at very short distances and in turn this allows us to probe the superimposed light-induced attractions, without particles sticking together irreversibly (see Supplementary Fig. 1). We follow the approach of Grier and coworkers^41^, to obtain an autocalibrated measurement of the colloidal interaction potential by analysing the differences between the distributions of the particle positions in the presence and the absence of optically induced forces (see Supplementary Methods and Supplementary Fig. 2). Thus, in our experiments, by turning on and off the random light field, we obtain directly the light-induced interaction potential without an explicit measurement of the complete pair interaction potential. Because of the finite width of the laser traps, the particles sample only a small range of *U* around *r*_0_. To determine the potential over a wider range of distances, we repeat the measurements for different relative positions of the optical traps (umbrella sampling). Typically, we start at an average trap separation that is near contact and increase *r*_0_ in steps of *Δ*=40 nm until six different trap positions are scanned. *Δ* is chosen to be smaller than the width of the trap potential 2*σ*≈140 nm, to have sufficient overlap between neighbouring trap separations.

**Figure 3:**
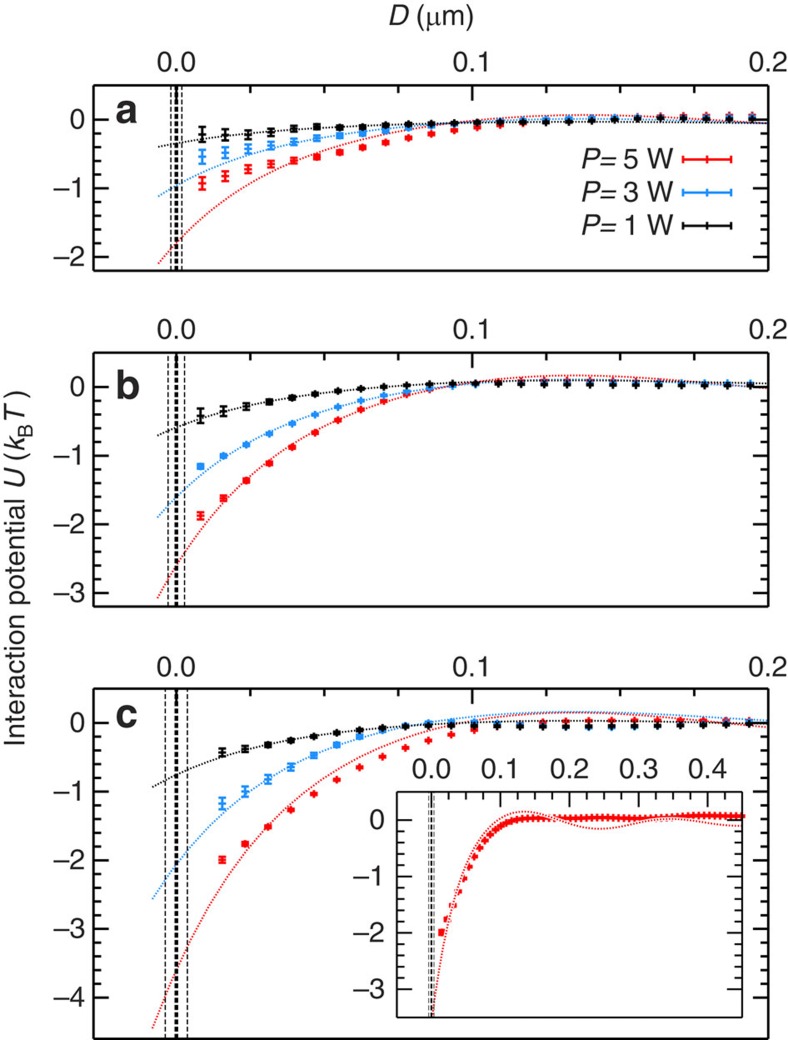
Optical binding between dielectric microspheres in random light fields. From the analysis of the thermal motion in the two adjacent optical traps, the particle interaction potential *U*(*D*) is obtained experimentally. The melamine (*n*=1.68) microspheres have sizes 2*R*=2 μm (**a**), 2*R*=3 μm (**b**) and 2*R*=4 μm (**c**). All particles are suspended in a 2.7 mM KCl aqueous solution. In the figure, the measured interaction potential is shown for three different laser power settings *P* in each panel. The vertical lines show the estimated contact positions and the experimental uncertainty Δ*D*∼±5 nm. By numerical evaluation of equation (6), we can fit the data and then extrapolate *U*(*D*; *P*) to obtain values for the contact potential *U*_0_ as a function of particle size 2*R* and laser power *P*. The inset shows an enlarged plot for 2*R*=4 μm and *P*=5 W. Notice that the depicted experimental graphs *U*(*D*) reflect an average of 16 independent measurements including s.d.

The final results for *U*(*D*=*r*−2*R*), shown in Fig. 3, are obtained by averaging over 16 independent experimental runs carried out under the same conditions. We find clear evidence for OB in a random light field. Moreover, we are able to quantify the potential *U*(*D*=*r*−2*R*) as well as its dependence on the incident light power. Varying the laser power from 5.0 to 1.0 W weakens the attractive potential. For an estimate of the contact potential *U*_0_≡*U*(*D*=0), we adjust the prefactor in equation (6) for a best fit to the data as shown in Fig. 3. The overall agreement between experiments and theory is remarkable, except for the predicted oscillation of the interaction potential at large interparticle distances. We believe the lack of clear oscillations can be explained by the approximations made when deriving equation (6) in the first Born (Rayleigh–Gans) approximation. The predicted oscillations are due to constructive and destructive interferences when summing over equidistant pairs of elementary dipolar scatters in the particle. However, for higher refractive indexes these interferences are smeared out, as equidistant pairs of dipoles positioned at different distances from the particle surface do not contribute in the same way anymore. An additional contribution that might affect the comparison between theory and experiment is the absence of a full 4*π* isotropic illumination in the experiment due to the absence of incoming photons with momentum near parallel to the mirror.

**Figure 4:**
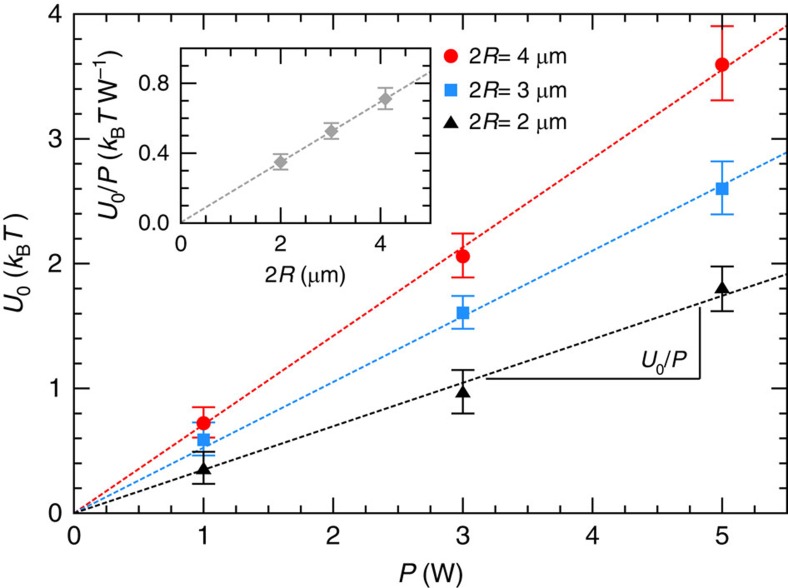
Laser power and particle size dependence of the contact potential. Contact potential *U*_0_ as a function of the laser power *P* for particles of different size 2*R*=2, 3 and 4 μm. *U*_0_ including its error has been obtained by extrapolating the experimental data depicted in Fig. 3. From a linear fit (dashed lines) we estimate the normalized contact potential *U*_0_/*P* for the respective particle sizes. The inset shows the dependence of *U*_0_/*P* on the particle diameter 2*R* and the dotted line is a linear fit to the data U_0_/*P*∼0.17 *k*_B_*T* W^−1^ × (2*R* μm^−1^).

In Fig. 4, we display results for the contact potential *U*_0_ as a function of laser power *P* for the three different particle sizes. From the slope of the linear fits (dashed lines) we can extract the normalized contact potential *U*_0_/*P*, which does not depend on the laser power. *U*_0_/*P* increases with particle size and the data set is consistent with the linear increase as predicted by equations (6) and (7). Finally, we attempt a quantitative comparison between the experimental results and the theoretical predictions for the contact potential *U*_0_. We note that such a comparison is based on a number of uncertainties related to the approximations made in the theory as well as the experiment. From equation (6), we compute the theoretically predicted values for melamine microspheres with an index of refraction of *n*_P_=1.68 in water (*n*_h_=1.33) as a function of particle size. For the cross-sectional area of the light-filled cavity, we use *A*≃0.004 mm^2^. Moreover, we take the light power in the cavity equal to the incident laser power for a vacuum-wavelength *λ*_0_=532 nm. Under these assumptions, the theory predicts 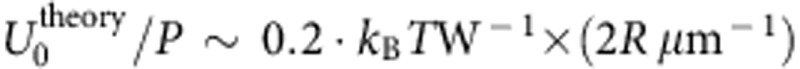. This result matches the experimental value 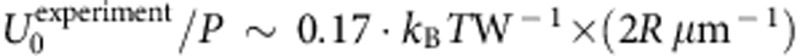 (Fig. 4, inset). Given the approximations made, such a near quantitative agreement might be somewhat fortuitous but nonetheless the overall agreement between experiment and theory supports our findings.

## Discussion

In this study, we introduce the concept of random-light-field-induced forces as a tool to control translationally invariant interactions between small particles in two or three dimensions with tuneable strength and range. Such external control over isotropic interactions in colloidal systems is unprecedented and of key importance^7^,^8^,^9^. Other ways to control colloidal interactions, such as adjusting the solvent quality, field-induced dipolar interactions or double-layer screening, are limited in scope and precision or involve the change of composition^8^,^9^,^10^,^11^. Nonetheless, these strategies are widely applied even though they do not provide the level of control over interaction forces that, as discussed here, can be achieved by using externally applied random light fields. Moreover, light-induced forces between particles act instantly on time scales of Brownian motion. This rapid response opens the path for new applications in three-dimensional colloidal systems: random-light-controlled forces of sufficient strength can induce phase separation and aggregation, but when applied dynamically could also be targeted to stabilize non-equillilibrium phases or to anneal defects in periodic structures such as photonic crystals. In summary, the approach presented here will open the pathway for a new field of fundamental research on isotropic optical forces and at the same time it will equip researcher with a powerful tool to control and manipulate the self-assembly and phase behaviour of colloidal particle assemblies on nano- and mesoscopic length scales^6^,^7^.

## Methods

### The creation of artificial random light fields

A focused green laser beam (Coherent Verdi-V5, *λ*=532 nm, calculated 1/*e*^2^ beam-waist 2*w*_0_=25 μm) with a laser power of up to 5 W is focused with an *f*=75 mm lens on the back surface of a glass capillary filled with a dense amorphous solid composed of PMMA (polymethylmethacrylate) beads, diameter ∼0.4 μm, with a layer thickness of ∼20 μm. This first layer scatters >99% of the incident power and thereby creates a random light field in the sample cavity of thickness *t*≃15 μm (inset, Fig. 2). The latter is filled with a very dilute suspension of melamine microspheres (Microparticles GmbH, Germany) with a diameter of 2 μm≤2*R*≤4 μm and refractive index *n*=1.68, dispersed in aqueous solution and sealed with ultraviolet-curable glue. To screen the electrostatic repulsions, we add 2.7 mM KCl. This results in a Debye screening length of *λ*_D_≃6 nm. The thickness of the layer containing the melamine microspheres is controlled by adding a small amount of *t*=2*R*=15 μm silica spheres that serve as spacers. Both layers are separated by the outer glass wall of the capillary with a thickness of *h*=20±2 μm (CM Scientific). A dichroic mirror with a reflectivity >99% at *λ*=532 nm (for incident angles 0°–45°, custom made by Asphericon GmbH, Jena, Germany) is placed on the opposite side of the cell. The mirror is transparent for wavelengths above *λ*∼650 nm, allowing both the trapping and the visual observation of the melamine microspheres. The clear layer sandwiched between the turbid layer and the mirror (cavity) thus has a thickness of *h*+*t*≃35 μm. The thin slab geometry together with the scattering in the turbid layer and multiple reflections in the cavity assure that in the centre of the sample cell we generate a fairly isotropic and quasi-monochromatic random light field. The volume speckle inside the cavity displays intensity fluctuations on a typical length scale^42^ of *λ*/2∼200 nm. In contrast with the anisotropic static light speckle pattern used to generate random potentials landscapes in the quest for Anderson localization of matter waves^43^, we generate random temporal fluctuations of the light fields by rapidly scanning the green laser focal spot over the surface of the turbid layer. We set the scan distance to ±7 μm at an oscillation frequency of 500 Hz using a galvano mirror. The oscillation frequency is chosen in a way that light field fluctuates randomly much faster than *τ*_B_=*R*^2^/(6*D*_0_)=776 ms, the time scale for Brownian motion of the smallest melamine microspheres under study. Here, *D*_0_ denotes the Brownian diffusion coefficient.

It has been recently reported that the collective motion of a large set of microspheres under the influence of both Brownian and self-interacting optical forces becomes active and their dynamical quantities are no longer representative of thermodynamic equilibrium^44^. In our case, however, the rapidly fluctuating speckle is not coupled to the motion of the melamine spheres. Other recent experimental studies have investigated small particles in one- and two-dimensional spatially modulated light fields^45^,^46^,^47^. Such static or slowly varying light fields, however, influence the behaviour of small particles in a fundamentally different way than dispersion forces. They result in a quasi-static potential energy landscapes similar to the case of optical traps. The same strategies have already been exploited earlier to create energy landscapes with crystalline or quasicrystalline order with the aim to manipulate or influence the spatial distribution of small particles in two dimensions^14^,^48^. In our case, however, the mean light power is spatially invariant on Brownian and longer times scales, which, in full analogy to dispersion forces, leads to translationally invariant interactions in contrast to these previous approaches.

It is important to note that roughly two thirds of the incident laser light will be reflected by the turbid layer. However, the reduced incident power is compensated by multiple reflections inside the cavity, except for the small residual losses by the dichroic mirror. Therefore, we expect the laser power in the cavity to be approximately the same as the incident laser power *P*. Reflections in the cavity can lead to an increase of the effective surface area *A* as the reflected light can spread out laterally. To obtain an estimate of *A*, we image the residual green light, for an incident laser power of 1 W, transmitted by the dichroic mirror by increasing the exposure time and the gain setting of the camera corresponding to an increase in detection efficiency by more than four orders of magnitude. From an analysis of the slightly elliptical intensity distribution, we derive an areal cross-section of *A*∼*π* × 41 × 34 μm^2^ ∼0.004 mm^2^ based on the 1/*e* decay length along the major and the minor axis. This means that for a laser power of up to 5 W, we can indeed reach intensities ∼mW μm^−2^ comparable to the case of optical tweezing.

### Experimental procedure

In one experimental run, we observe the Brownian motion of two micron-sized melamine microspheres that are held at a mean distance *r*_0_ in the centre of the light-filled cavity, both laterally and axially, using a specifically adapted Nikon Eclipse TS100 bright-field microscope composed of (i) a long working distance objective (20 × /0.42 EO Plan Apo ELWD) for the sidewise white light illumination, (ii) an oil-immersion objective for both the trapping and the observation of the particle motion using a CCD (charge-coupled device) camera and (iii) a notch filter to filter out residual stray light coming from the trapping laser as well as a dielectric mirror to couple the trapping laser into the optical path of the microscope.It is noteworthy that the bright-field illumination takes place across the first diffusing layer. For the laser trapping, we use a near-infrared laser beam (Toptica DL 100) with a wavelength of 785 nm and a power of ∼8 mW (measured at the exit of the laser) that is rapidly switched between two positions with a galvano mirror to produce the time shared dual traps^37^,^38^,^39^.

Using a digital camera (Prosilica GC650, Allied Vision Technologies GmbH, Germany) we record images of 120 × 120 pixels with a frame rate of 90 Hz and an exposure time of 0.3 ms at × 80 magnification. The edge length per pixel is *d*_pix_≈100 nm, which provides a sub-pixel localization accuracy of the particle centre better than 10 nm^41^. For a given mean separation distance *r*_0_, we perform two experiments (see Fig. 2). In the first experiment, we acquire a movie of 4,000 images at a frame rate of 90 Hz under the influence of a random light field. In a subsequent reference experiment, the random light field is turned off and the measurement is repeated under otherwise identical conditions. The recorded sequence of images is analysed using a standard particle-tracking algorithm^49^ to obtain distributions of the centre-to-centre separations of the trapped particles for both experiments (see Supplementary Methods and Supplementary Fig. 2).

## Additional information

**How to cite this article**: Brügger, G. *et al.* Controlling dispersion forces between small particles with artificially created random light fields. *Nat. Commun.* 6:7460 doi: 10.1038/ncomms8460 (2015).

## Supplementary Material

Supplementary Information Supplementary Figures 1-2, Supplementary Notes 1-6, Supplementary Methods and Supplementary References.
