# Supplementary material for: Controlling dispersion forces between small particles with artificially created random light fields
Source: Nat Commun. 2015 Jun 22;6:7460. doi: 10.1038/ncomms8460 (PMC4557368; doi:10.1038/ncomms8460)
Supplement: Supplementary Information — Supplementary Figures 1-2, Supplementary Notes 1-6, Supplementary Methods and Supplementary References. [file ncomms8460-s1.pdf]

Supplementary Figures

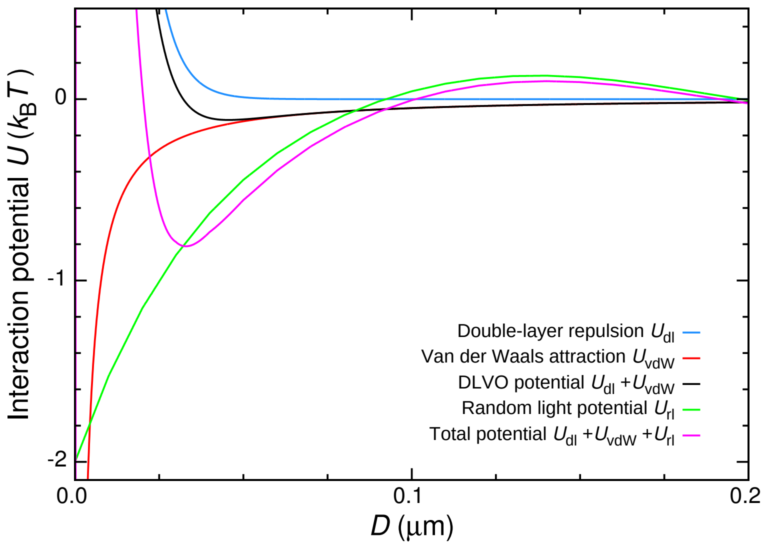

Supplementary Figure 1: **Random Light Induced and DLVO interaction potentials**. Calculated interaction potential for charge stabilized colloidal particles of size $2R = 2$ µm suspended in water in the presence and the absence of the random light (rl) induced interactions. The combination of van der Waals attractions (vdW - red line), Hamaker constant $A = 0.1\ k_{\mathrm{B}}T$, and electrostatic double layer repulsions (dl-blue line), Debye length $\lambda_{\mathrm{D}} = 6$ nm, contact potential $U_{\mathrm{dl}}(D = 0) = 44\ k_{\mathrm{B}}T$ leads to the DLVO potential (black line). The attractive potential due to random light forces (green line), monochromatic random illumination with a contact potential of -2 $k_{\mathrm{B}}T$, equation (6) is superimposed leading to the total potential (magenta line). The repulsive part dominates at very short distances and in turn this allows us to probe the superimposed light induced attractions, without particles sticking together irreversibly.

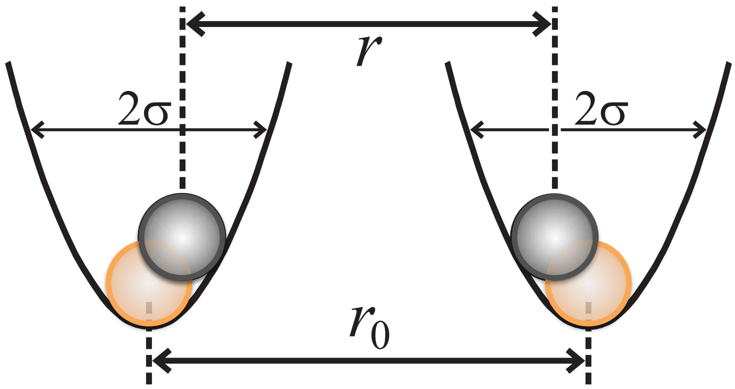

Supplementary Figure 2: **Thermal motion inside adjacent optical traps.** Two particles with radius R inside two identical optical traps are positioned at a distance r_0. For an isolated particle the distribution of positions is Gaussian with a standard deviation σ, which is set by the trap stiffness. Thus 2σ is a measure for the typical distances $r - r_0$ probed by thermal motion. Interactions between particles lead to a characteristic change in the distribution of particle positions. Attractive interactions increase the probability for the particles to approach. Precise measurements of $f_{\text{pair}}(r)$ are therefore a sensitive tool to determine the particle-particle interaction potential $U(D = r - 2R)$.

Supplementary Notes

Supplementary Note 1: Field-Field correlations and cross-spectral density in a stationary random field

We consider a fluctuating electric field, $\mathbf{E}(\mathbf{r}, t)$ in a transparent and non-dispersive homogeneous medium with real refractive index $n_\mathrm{h} = \sqrt{\epsilon_\mathrm{h}}$. For a stationary field[1], the spatiotemporal fluctuations,

$$\langle E_i(\mathbf{r}, t) E_j(\mathbf{r}', t')\rangle = \mathrm{Re}\left\{\int_0^\infty \frac{\mathrm{d}\omega}{2\pi} W_{ij}(\mathbf{r}, \mathbf{r}',\ \omega) e^{-i\omega(t-t')}\right\}, \tag{1}$$

are characterized by the the cross-spectral density tensor $W_{ij}(\mathbf{r}, \mathbf{r}',\ \omega)$ given by[2]

$$W_{ij}(\mathbf{r}, \mathbf{r}',\ \omega)\ \equiv\ \langle E_i^*(\mathbf{r}, \omega) E_j(\mathbf{r}', \omega)\rangle = \frac{4\pi}{\epsilon_0\epsilon_\mathrm{h}} u_\mathrm{E}(\mathbf{r}, \omega)\ \left\{\frac{2\pi}{k}\mathrm{Im}\{G_{ij}(\mathbf{r}, \mathbf{r}',\ \omega)\}\right\} \tag{2}$$

where $G_{ij}(\mathbf{r}, \mathbf{r}',\ \omega)$ are the matrix elements of the Green tensor,

$$\overleftrightarrow{\mathbf{G}}(\mathbf{r}, \mathbf{r}') = \frac{k}{4\pi}\left[\mathbf{I}_3 + \frac{1}{k^2}\boldsymbol{\nabla}\boldsymbol{\nabla}\right]\frac{\exp(ik|\mathbf{r}-\mathbf{r}'|)}{k|\mathbf{r}-\mathbf{r}'|}, \tag{3}$$

($\mathbf{I}_3$ is the identity tensor) and $u_\mathrm{E}(\mathbf{r}, \omega)$ is related to the time-averaged electric energy per unit volume,

$$\langle U(\mathbf{r}, t)\rangle = \frac{\epsilon_\mathrm{h}\epsilon_0}{2}\langle|\mathbf{E}(\mathbf{r}, t)|^2\rangle = \int_0^\infty u_\mathrm{E}(\omega)\mathrm{d}\omega. \tag{4}$$

Supplementary Note 2: Multiple scattering between two compact bodies

We consider a particle A, centered at the origin of coordinates, and a particle B displaced a distance r along the positive z-axis. From a physical point of view, instead of a continuous approach, each particle can be seen as made of discretized, N_A and N_B, identical cubic elements of volume v. This is also known as a discrete dipole approach (DDA)[3]. In the presence of an external polarizing field, $\mathbf{E}_\mathrm{inc}(\mathbf{r}, \omega)$, each volume element acts as an induced dipole proportional to the polarizing field, i.e.

$$\mathbf{p}(\mathbf{r}_n, \omega) = \epsilon_0\epsilon_\mathrm{h}\ \alpha(\omega)\mathbf{E}_\mathrm{inc}(\mathbf{r}_n, \omega) \tag{5}$$

where $\alpha(\omega)$ is the polarizability which, for cubic or spherical elements of volume v, is given by[4]

$$\alpha(\omega) \equiv \frac{v\tilde{\alpha}_0}{1 - i\frac{vk^3}{6\pi}\tilde{\alpha}_0}\quad,\quad \tilde{\alpha}_0(\omega) \equiv 3\frac{\epsilon(\omega)-\epsilon_\mathrm{h}}{\epsilon(\omega)+2\epsilon_\mathrm{h}}. \tag{6}$$

The polarizing field on a given element in particle B, $\mathbf{E}_{\text{inc}}(\mathbf{r}_n^{\mathrm{B}},\omega)$, is given by the solution of the multiple scattering problem:

$$\mathbf{E}_{\text{inc}}(\mathbf{r}_n^{\mathrm{B}}) \;=\; \mathbf{E}_0(\mathbf{r}_n^{\mathrm{B}}) + \alpha_{\mathrm{B}}k^2\sum_{m\neq n}^{N_{\mathrm{B}}}\overleftrightarrow{\mathbf{G}}(\mathbf{r}_n^{\mathrm{B}},\mathbf{r}_m^{\mathrm{B}})\mathbf{E}_{\text{inc}}(\mathbf{r}_m^{\mathrm{B}}) + \alpha_{\mathrm{A}}k^2\sum_{m}^{N_{\mathrm{A}}}\overleftrightarrow{\mathbf{G}}(\mathbf{r}_n^{\mathrm{B}},\mathbf{r}_m^{\mathrm{A}})\mathbf{E}_{\text{inc}}(\mathbf{r}_m^{\mathrm{A}}) \quad (7)$$

(and an equivalent equation for particle A). For simplicity in the notation, we do not include here the explicit ω-dependence. These are a set of $3N_{\mathrm{A}}+3N_{\mathrm{B}}$ equations that can be written in compact matrix form as

$$\mathbf{E}_{\text{inc}}(\mathrm{B}) \;=\; \mathbf{E}_0(\mathrm{B}) + \alpha_{\mathrm{B}}k^2\overleftrightarrow{\mathbf{G}}_{\mathrm{B,B}}\mathbf{E}_{\text{inc}}(\mathrm{B}) + \alpha_{\mathrm{A}}k^2\overleftrightarrow{\mathbf{G}}_{\mathrm{B,A}}\mathbf{E}_{\text{inc}}(\mathrm{A}) \quad (8)$$

$$\mathbf{E}_{\text{inc}}(\mathrm{A}) \;=\; \mathbf{E}_0(\mathrm{A}) + \alpha_{\mathrm{A}}k^2\overleftrightarrow{\mathbf{G}}_{\mathrm{A,A}}\mathbf{E}_{\text{inc}}(\mathrm{A}) + \alpha_{\mathrm{B}}k^2\overleftrightarrow{\mathbf{G}}_{\mathrm{A,B}}\mathbf{E}_{\text{inc}}(\mathrm{B}). \quad (9)$$

Introducing the $\mathbf{T}$-matrix, defined as

$$\mathbf{T}^{-1}(\mathbf{r}_n^{\mathrm{B}},\mathbf{r}_m^{\mathrm{B}}) = \frac{1}{\alpha_{\mathrm{B}}k^2}\mathbf{I}_3 - \overleftrightarrow{\mathbf{G}}(\mathbf{r}_n^{\mathrm{B}},\mathbf{r}_m^{\mathrm{B}})(1-\delta_{nm}), \quad \text{or} \quad \mathbf{T}_{\mathrm{B}}^{-1} \equiv \frac{1}{\alpha_{\mathrm{B}}k^2}\mathbf{I}_{3N_{\mathrm{B}}} - \overleftrightarrow{\mathbf{G}}_{\mathrm{B,B}} \quad (10)$$

where $\mathbf{I}_{3N_{\mathrm{B}}}$ is the $3N_{\mathrm{B}}\times 3N_{\mathrm{B}}$ identity matrix (and an equivalent expression for $\mathbf{T}_{\mathrm{A}}$), the formal solution of the scattering problem can be written as

$$\mathbf{E}_{\text{inc}}(\mathrm{B}) \;=\; \frac{1}{\alpha_{\mathrm{B}}k^2}\left(\mathbf{T}_{\mathrm{B}}^{-1} - \overleftrightarrow{\mathbf{G}}_{\mathrm{B,A}}\mathbf{T}_{\mathrm{A}}\overleftrightarrow{\mathbf{G}}_{\mathrm{A,B}}\right)^{-1}\left(\mathbf{E}_0(\mathrm{B}) + \overleftrightarrow{\mathbf{G}}_{\mathrm{B,A}}\mathbf{T}_{\mathrm{A}}\mathbf{E}_0(\mathrm{A})\right) \quad (11)$$

$$\mathbf{E}_{\text{inc}}(\mathrm{A}) \;=\; \frac{1}{\alpha_{\mathrm{A}}k^2}\left(\mathbf{T}_{\mathrm{A}}^{-1} - \overleftrightarrow{\mathbf{G}}_{\mathrm{A,B}}\mathbf{T}_{\mathrm{B}}\overleftrightarrow{\mathbf{G}}_{\mathrm{B,A}}\right)^{-1}\left(\mathbf{E}_0(\mathrm{A}) + \overleftrightarrow{\mathbf{G}}_{\mathrm{A,B}}\mathbf{T}_{\mathrm{B}}\mathbf{E}_0(\mathrm{B})\right). \quad (12)$$

Our approach can be seen as the DDA-like version of the well known $\mathbf{T}$-matrix approach of multiple scattering of electromagnetic waves by two different objects usually described in terms of a basis of multipolar vector wave functions (see for example refs 5,6).

Supplementary Note 3: Optical interactions between two compact bodies induced by random light fields

In the presence of a random (stationary) field, $\mathbf{E}_0(\mathbf{r},t)$, both the dipoles and the polarizing fields are, in general, fluctuating quantities and the time averaged force along the z-axis may be written as the sum of two different terms (see for example, ref. 7)

$$F_z = \left\langle \mathbf{p}^{\text{ind}}(\mathbf{r}_n,t)\,\frac{\partial}{\partial z}\mathbf{E}_{\text{inc}}^{\text{fluc}}(\mathbf{r},t)\Big|_{\mathbf{r}=\mathbf{r}_n}\right\rangle + \left\langle \mathbf{p}^{\text{fluc}}(t)\,\frac{\partial}{\partial z}\mathbf{E}_{\text{inc}}^{\text{ind}}(\mathbf{r},t)\Big|_{\mathbf{r}=\mathbf{r}_n}\right\rangle \quad (13)$$

where the first term describes the force induced by the fluctuating (external) field, $\mathbf{E}_0^{\text{fluc}}$ with the corresponding induced dipole $\mathbf{p}^{\text{ind}}$ as discussed in the main text. The second involves the (spontaneous and thermal) fluctuations of the dipole $\mathbf{p}^{\text{fluc}}$.

We focus on lossless particles and discard the second contribution (in absence of absorption, there are no spontaneous and thermal fluctuations of the dipoles). From equations (5) and (13), the total time-averaged force on particle B is then given by

$$F_z^{\mathrm{B}} = \epsilon_0\epsilon_{\mathrm{h}}\mathrm{Re}\left\{\int_0^\infty \frac{\mathrm{d}\omega}{2\pi}\alpha_{\mathrm{B}}(\omega)\sum_n^{N_{\mathrm{B}}}\left\langle \mathbf{E}_{\mathrm{inc}}(\mathbf{r}_n^{\mathrm{B}},\omega).\,\frac{\partial}{\partial z}\mathbf{E}_{\mathrm{inc}}^*(\mathbf{r},\omega)\Big|_{\mathbf{r}=\mathbf{r}_n^{\mathrm{B}}}\right\rangle\right\} \tag{14}$$

where $\mathbf{E}_{\mathrm{inc}}(\mathbf{r}_n,\omega)$ is given in equation (7) and the gradient of the incoming field is the sum of three different terms

$$\begin{aligned}\frac{\partial}{\partial z}\mathbf{E}_{\mathrm{inc}}(\mathbf{r},\omega)\Big|_{\mathbf{r}=\mathbf{r}_n^{\mathrm{B}}} &= \frac{\partial}{\partial z}\mathbf{E}_0(\mathbf{r})\Big|_{\mathbf{r}=\mathbf{r}_n^{\mathrm{B}}} + \alpha_{\mathrm{A}}k^2\sum_m^{N_{\mathrm{A}}}\frac{\partial}{\partial z}\overleftrightarrow{\mathbf{G}}(\mathbf{r},\mathbf{r}_m^{\mathrm{A}})\mathbf{E}_{\mathrm{inc}}(\mathbf{r}_m^{\mathrm{A}})\Big|_{\mathbf{r}=\mathbf{r}_n^{\mathrm{B}}}\\ &\quad+\alpha_{\mathrm{B}}k^2\sum_{m\neq n}^{N_{\mathrm{B}}}\frac{\partial}{\partial z}\overleftrightarrow{\mathbf{G}}(\mathbf{r},\mathbf{r}_m^{\mathrm{B}})\mathbf{E}_{\mathrm{inc}}(\mathbf{r}_m^{\mathrm{B}})\Big|_{\mathbf{r}=\mathbf{r}_n^{\mathrm{B}}}.\end{aligned} \tag{15}$$

These three terms give three different contributions to the total force on particle B. The first term, F_z^{B1}, can be seen as coming from the homogeneous radiation field on particle B (which after arriving at B, suffers multiple scattering events with particle A). The second, F_z^{B2}, comes from the radiation first scattered by particle A. The last term, arising from the multiple scattering interactions inside the particle, does not contribute to the total force on B since these interactions cancel out when summing over all the dipoles in B after averaging over the random field. Taking into account that

$$\left\langle E_{0j}(\mathbf{r}_n^{\mathrm{A}},\omega)\,\frac{\partial}{\partial z}\mathbf{E}_{0i}^*(\mathbf{r},\omega)\Big|_{\mathbf{r}=\mathbf{r}_m^{\mathrm{B}}}\right\rangle = \frac{u_{\mathrm{E}}(\omega)}{\epsilon_0\epsilon_{\mathrm{h}}}\frac{8\pi^2}{k}\mathrm{Im}\left(\frac{\partial}{\partial z}\{G_{ij}(\mathbf{r},\mathbf{r}_n^{\mathrm{A}},\,\omega)\}\right)_{\mathbf{r}=\mathbf{r}_m^{\mathrm{B}}} \tag{16}$$

$$\left(\frac{\partial}{\partial z}\{\overleftrightarrow{\mathbf{G}}(\mathbf{r},\mathbf{r}_n^{\mathrm{A}},\,\omega)\}\right)_{\mathbf{r}=\mathbf{r}_m^{\mathrm{B}}} = \frac{\partial}{\partial r}\{\overleftrightarrow{\mathbf{G}}(\mathbf{r}_m^{\mathrm{B}}-\mathbf{r}_n^{\mathrm{A}},\,\omega)\} \tag{17}$$

we find $F_z^{\mathrm{B}} = \int_0^\infty \mathrm{d}\omega[F_z^{\mathrm{B1}}(\omega)+F_z^{\mathrm{B2}}(\omega)]$ with

$$F_z^{\mathrm{B1}}(\omega) = \frac{4\pi u_{\mathrm{E}}(\omega)}{k^3}\mathrm{Tr}\left[\mathrm{Im}\left\{\frac{\partial}{\partial r}\overleftrightarrow{\mathbf{G}}_{\mathrm{B,A}}\right\}\mathrm{Re}\left\{\mathbf{T}_{\mathrm{A}}\overleftrightarrow{\mathbf{G}}_{\mathrm{A,B}}\left(\mathbf{T}_{\mathrm{B}}^{-1}-\overleftrightarrow{\mathbf{G}}_{\mathrm{B,A}}\mathbf{T}_{\mathrm{A}}\overleftrightarrow{\mathbf{G}}_{\mathrm{A,B}}\right)^{-1}\right\}\right] \tag{18}$$

where "Tr" stands for the trace of the $3N_{\mathrm{B}}\times 3N_{\mathrm{B}}$ matrix. After some algebra and taking into account that in absence of absorption (i.e. $\epsilon(\omega)$ and $\tilde{\alpha}_0$ are real)

$$\mathrm{Im}\mathbf{T}_{\mathrm{B}}^{-1} \equiv \frac{k}{6\pi}\mathbf{I}_{3N_{\mathrm{B}}} - \mathrm{Im}\overleftrightarrow{\mathbf{G}}_{\mathrm{B,B}}\,,\quad \mathrm{Im}\mathbf{T}^{-1}(\mathbf{r}_n^{\mathrm{B}},\mathbf{r}_m^{\mathrm{B}}) = -\mathrm{Im}\overleftrightarrow{\mathbf{G}}(\mathbf{r}_n^{\mathrm{B}},\mathbf{r}_m^{\mathrm{B}}), \tag{19}$$

the second contribution can be shown to be given by

$$F_z^{\rm B2}(\omega) \;=\; \frac{4\pi u_{\rm E}(\omega)}{k^3}{\rm Tr}\left[{\rm Re}\left\{\frac{\partial}{\partial r}\overleftrightarrow{\mathbf{G}}_{\rm B,A}\right\}{\rm Im}\left\{\mathbf{T}_{\rm A}\overleftrightarrow{\mathbf{G}}_{\rm A,B}\left(\mathbf{T}_{\rm B}^{-1}-\overleftrightarrow{\mathbf{G}}_{\rm B,A}\mathbf{T}_{\rm A}\overleftrightarrow{\mathbf{G}}_{\rm A,B}\right)^{-1}\right\}\right]. \quad (20)$$

Adding equations (18) and (20) we finally obtain that, in absence of absorption, the total force is conservative $F_z^{\rm B} = -\partial U(r)/\partial r$ with an interaction potential given by

$$U(r) = \int_0^\infty {\rm d}\omega\; \frac{2\pi}{k^3}u_{\rm E}(\omega)\;{\rm ImTr}\left[\ln\left(\mathbf{I}-\overleftrightarrow{\mathbf{G}}_{\rm B,A}\mathbf{T}_{\rm A}\overleftrightarrow{\mathbf{G}}_{\rm A,B}\mathbf{T}_{\rm B}\right)\right]. \quad (21)$$

The dependence of the interaction on distance is completely contained in $\overleftrightarrow{\mathbf{G}}_{\rm B,A}$ whereas all the shape and material dependence is contained in the $\mathbf{T}$-matrices.

In the case of equilibrium thermal blackbody radiation the electric energy density, $U_{\rm E}(\omega)$, is given by[1,8]

$$u_{\rm E}(\omega){\rm d}\omega = \frac{\hbar\omega}{2}\coth\left(\frac{\hbar\omega}{2k_{\rm B}T}\right)\frac{n_{\rm h}^3\omega^2}{2\pi^2c^3}{\rm d}\omega \quad (22)$$

which, at zero temperature gives $u_{\rm E}(\omega) = \hbar k^3/(4\pi^2)$. For absorbing (emitting) particles in equilibrium, we can include in equation (13) the contribution of the fluctuating dipoles and the corresponding radiated fields[7] (linked through the fluctuation-dissipation theorem). Interestingly, in equilibrium, this additional contribution conspires with the force due to the field fluctuations to give a total interaction potential which is exactly given by equation (21), now including light absorption and emission (i.e. ${\rm Im}\{\epsilon(\omega)\} \geq 0$) and we recover the exact Casimir interaction between arbitrary compact objects[9,10].

Supplementary Note 4: Attractive and repulsive interactions between dipolar electric and magnetic particles

Submicron dielectric spheres made of moderate permittivity materials present dipolar magnetic and electric responses[11], characterized by their respective first-order "Mie" coefficients, in the near infrared, in such a way that either of them can be selected by choosing the illumination wavelength. The scattering properties of Silicon and other semiconductor nanoparticles[11], can be well described by their electric and magnetic polarizabilities, being negligible the contribution of higher order modes (contribution of higher order modes can be relevant when the interparticle distance D becomes of the order of the particle radius; a detailed analysis of these interactions will be described elsewhere). When the optical response of the particles can be described by their

electric and magnetic polarizabilities, $\alpha_n^{\rm e}(\omega)$ and $\alpha_n^{\rm m}(\omega)$ respectively ($n = {\rm A}, {\rm B}$). The presence of an external polarizing field induce both electric, $\mathbf{p}$ and magnetic, $\mathbf{m}$, dipoles, i.e.

$$\mathbf{p}(\mathbf{r}_n,\omega) \;=\; \epsilon_0\epsilon_{\rm h}\,\alpha_n^{\rm e}(\omega)\mathbf{E}_{\rm inc}(\mathbf{r}_n,\omega) \tag{23}$$

$$\mathbf{m}(\mathbf{r}_n,\omega) \;=\; \alpha_n^{\rm m}(\omega)\mathbf{H}_{\rm inc}(\mathbf{r}_n,\omega) = -\frac{i\alpha_n^{\rm m}(\omega)}{kZ}\,\boldsymbol{\nabla}\times\mathbf{E}_{\rm inc}(\mathbf{r},\omega)\big|_{\mathbf{r}=\mathbf{r}_n} \tag{24}$$

where $Z \equiv \sqrt{\mu_0/(\epsilon_0\epsilon_{\rm h})}$ is the impedance of the homogeneous medium. The polarizabilities are simply related to the first electric, a_1, and magnetic, b_1 Mie coefficients:

$$\alpha_n^{\rm e}(\omega) = i\frac{6\pi}{k^3}a_1\,, \quad \alpha_n^{\rm m}(\omega) = i\frac{6\pi}{k^3}b_1. \tag{25}$$

When we just consider dipolar particles we can write

$$\mathbf{T}_{\rm B} = k^2\begin{pmatrix}\alpha_{\rm B}^{\rm e}\mathbf{I} & 0\\ 0 & \alpha_{\rm B}^{\rm m}\mathbf{I}\end{pmatrix} = \begin{pmatrix}T_{\rm B}^{\rm e}\mathbf{I} & 0\\ 0 & T_{\rm B}^{\rm m}\mathbf{I}\end{pmatrix} \quad \text{and} \quad \overleftrightarrow{\mathbf{G}}_{\rm BA} \equiv \begin{pmatrix}\overleftrightarrow{\mathbf{G}}_{\rm E}({\rm B},{\rm A}) & \overleftrightarrow{\mathbf{G}}_{\rm M}({\rm B},{\rm A})\\ \overleftrightarrow{\mathbf{G}}_{\rm M}({\rm B},{\rm A}) & \overleftrightarrow{\mathbf{G}}_{\rm E}({\rm B},{\rm A})\end{pmatrix} \tag{26}$$

with

$$\overleftrightarrow{\mathbf{G}}_{\rm E}({\rm B},{\rm A}) = \begin{pmatrix}G_{{\rm E},x} & 0 & 0\\ 0 & G_{{\rm E},x} & 0\\ 0 & 0 & G_{{\rm E},z}\end{pmatrix}, \quad \overleftrightarrow{\mathbf{G}}_{\rm M}({\rm B},{\rm A}) = \begin{pmatrix}0 & -G_{\rm M} & 0\\ G_{\rm M} & 0 & 0\\ 0 & 0 & 0\end{pmatrix} \tag{27}$$

being

$$G_{{\rm E},x}(r) \;=\; G_{{\rm E},y}(r) = \left(1+\frac{i}{kr}-\frac{1}{k^2r^2}\right)g(r) \tag{28}$$

$$G_{{\rm E},z}(r) \;=\; \left(-\frac{2i}{kr}+\frac{2}{k^2r^2}\right)g(r) \tag{29}$$

$$G_{\rm M}(r) \;=\; \left(i-\frac{1}{kr}\right)g(r). \tag{30}$$

and $g(r) = e^{ikr}/(4\pi r)$ the scalar Green function.

In absence of absorption the trace formula for the interaction potential [equation (21)] can be calculated in closed form as:

$$\begin{aligned}\mathrm{Tr}\left[\ln\left(\mathbf{I}-\overleftrightarrow{\mathbf{G}}_{\rm B,A}\mathbf{T}_{\rm A}\overleftrightarrow{\mathbf{G}}_{\rm A,B}\mathbf{T}_{\rm B}\right)\right] \;&=\\ =\ln\left(1-T_{\rm B}^{\rm e}T_{\rm A}^{\rm e}G_{{\rm E}z}^2(r)\right) \;&+\; \ln\left(1-T_{\rm B}^{\rm m}T_{\rm A}^{\rm m}G_{{\rm E}z}^2(r)\right)\\ +2\ln\Bigg[\left(1-T_{\rm B}^{\rm e}\left(T_{\rm A}^{\rm e}G_{{\rm E}x}^2(r)+T_{\rm A}^{\rm m}G_{\rm M}^2(r)\right)\right) &\left(1-T_{\rm B}^{\rm m}\left(T_{\rm A}^{\rm m}G_{{\rm E}x}^2(r)+T_{\rm A}^{\rm e}G_{\rm M}^2(r)\right)\right)\\ &-T_{\rm B}^{\rm e}T_{\rm B}^{\rm m}\left(T_{\rm A}^{\rm m}-T_{\rm A}^{\rm e}\right)^2G_{{\rm E}x}^2(r)G_{\rm M}^2(r)\Bigg].\end{aligned} \tag{31}$$

As shown in Fig. 2 in the main text, for two identical particles near the first Mie dipolar magnetic resonance the interaction force can be repulsive in analogy with the repulsive interactions between resonant atoms[12].

Supplementary Note 5: Random light forces between dipolar electric particles: gravitational like interactions

If we consider the long wavelength limit, where the magnetic polarizability is negligible, equation (21) takes the simple form

$$U(r) = \frac{2\pi}{k^3} U_{\mathrm{E}}(\omega) \, \mathrm{Im} \left\{ \sum_{i=x,y,z} \ln \left(\left[1 - \left(\alpha^{\mathrm{e}} k^2 G_{ii}(r) \right)^2 \right] \right) \right\} \tag{32}$$

which can be shown to be equivalent to equation (11) in ref. 13 in absence of absorption.

A remarkable prediction concerning optically induced interactions between atoms, molecules or small dipolar particles[14] is that, after averaging over all orientations of the inter-atomic axis with respect to the incident beam, the interaction is an isotropic long-range, "gravitational-like", $1/r$ potential in the near field. It was suggested[15] that this averaging could be experimentally achieved by an isotropic external illumination by means of multiple incoherent beams which, for atomic systems, could give rise to stable Bose-Einstein condensates with unique static properties[15]. An alternative is to average over all orientations and polarizations of the incoming, uncorrelated, plane waves[13]: In the weak scattering limit, expanding equation (32) leads to

$$U(r) \approx -\frac{2\pi}{k^3} U_{\mathrm{E}}(\omega) \, \mathrm{Im} \left\{ \sum_{i=x,y,z} \left(\alpha^{\mathrm{e}} k^2 G_{ii}(r) \right)^2 \right\} \tag{33}$$

which in the short distance limit gives the above mentioned long-range $1/r$ dependence of the optical interaction potential in agreement with previous results[13,14]. It is worth noticing that similar ideas were considered in the earlier proposal by Spitzer[16] of the so-called mock gravity, gravity-like interactions between matter in the universe due to background isotropic radiation pressure.

Supplementary Note 6: Weak scattering approximation

In the weak scattering limit, we can expand equation (21)

$$
\begin{aligned}
U(r) &\approx -\int_0^\infty \mathrm{d}\omega\, u_\mathrm{E}(\omega)\, 2\pi k\, \tilde{\alpha}_0^2 v^2 \mathrm{Im\ Tr}\left[\overleftrightarrow{\mathbf{G}}_{\mathrm{B,A}} \overleftrightarrow{\mathbf{G}}_{\mathrm{A,B}}\right] + \mathcal{O}(\tilde{\alpha}_0^3) && (34)\\
&= -\int_0^\infty \mathrm{d}\omega\, u_\mathrm{E}(\omega)\, 2\pi k\, \tilde{\alpha}_0^2 v^2 \sum_n^{N_\mathrm{B}} \sum_m^{N_\mathrm{A}} \sum_{i,j} \mathrm{Im}\left[G_{ij}^2(\mathbf{r}_n^\mathrm{B} - \mathbf{r}_m^\mathrm{A})\right] && (35)\\
&= -\int_0^\infty \mathrm{d}\omega\, u_\mathrm{E}(\omega)\, 2\pi k\, \tilde{\alpha}_0^2 \int_\mathrm{B} \mathrm{d}\mathbf{r}_\mathrm{B}^3 \int_\mathrm{A} \mathrm{d}\mathbf{r}_\mathrm{A}^3 \sum_{i,j} \mathrm{Im}\left[G_{ij}^2(|\mathbf{r}_\mathrm{B} - \mathbf{r}_\mathrm{A}|)\right]. && (36)
\end{aligned}
$$

It is easy to see that this is the result obtained at lowest order in [the so-called Born (or Rayleigh-Gans-Debye) approximation]. For two identical spheres of radius R, their centers being a distance r apart, in a quasi monochromatic random field, the interaction energy in the weak scattering limit can be seen as a Hamaker's integral[17]

$$
\begin{aligned}
U(D) &= -K \times \mathcal{U}(D, R, k)\\
\mathcal{U}(D, R, k) &= \frac{\pi^2}{r} \int_{r-R}^{r+R} \mathrm{d}y \left[\left(R^2 - (r-y)^2\right) \int_{y-R}^{y+R} \mathrm{d}x \Big\{\left(R^2 - (y-x)^2\right) x\, f(x)\Big\}\right] && (37)\\
f(x) &= \left(\frac{4\pi}{k}\right)^2 \mathrm{Im}\left\{\sum_{i,j} G_{ji}^2(x)\right\}\\
&= \mathrm{Im}\left\{e^{2ikx}\left(\frac{2}{(kx)^2} + \frac{4i}{(kx)^3} - \frac{10}{(kx)^4} - \frac{12i}{(kx)^5} + \frac{6}{(kx)^6}\right)\right\} && (38)
\end{aligned}
$$

with $D = r - 2R$ and $K = \{\mathrm{d}\omega\ 2u_\mathrm{E}(\omega)\}\pi k^3 \left[\tilde{\alpha}_0/(4\pi)\right]^2$.

Supplementary Methods

Laser trapping experiment and data treatment

The focused beam of a Topica DL 100 diode laser operating at a wavelength of 785 nm is time shared between two points using a galvano mirror (Galvoline G1432) driven by a square-wave oscillation at a frequency of 500 Hz. A telescope (Thorlabs, 3x Galilean optical beam expander, BE03M-B) is used to match the beam size with the back aperture of an oil immersion objective (Nikon 60x PlanApoVC, N.A.$= 1.4$)[18]. The location of the telescope is chosen in a way that the back focal plane of the oil immersion objective is imaged onto the galvano mirror, which allows for identical dual-traps[19]. Finally the time shared beam is focussed into the water layer of the sample cell to form the dual-trap. The particles are trapped in the middle of the water layer to minimize wall effects. The average distance between traps' centers can be changed by adjusting the amplitude of the galvano mirror oscillations. With the CCD camera we record images of 120×120 pixels with a frame rate of 90 Hz and an exposure time of 0.3 ms. With a micro-scale the effective pixel size is measured to be $d_{\text{pix}} \approx 0.1$ µm. The recorded images are analysed using an adapted MATLAB (The MathWorks, Inc., USA) code based on the particle tracking algorithm by Crocker and Grier[20] to finally obtain the particle positions on each picture. We quantify the transversal instrumental resolution of our apparatus by tracking two $2R = 2$ µm particles with a center-to-center separation of r_{s} that are permanently adsorbed to the lower glass surface of the water layer. A Gaussian fit to the measured distribution reveals a standard deviation of $\sigma_{xy} = 0.077$ pixel $= 7.7$ nm reflecting the transversal instrumental resolution. Moreover we have verified that out-of-plane fluctuations due to the finite trapping strength are negligible in our experiment[21].

The thermal motion of the two particles in the adjacent traps is illustrated in Supplementary Figure 2. For a given mean separation distance r_0 of the time shared optical traps we perform two experiments (see Fig. 1 of main text). In a first experiment we acquire a movie of 4000 images at a frame rate of 90 Hz under the influence of a random light field. In a subsequent reference experiment the random light field is turned off and the measurement is repeated under otherwise identical conditions. The recorded sequence of images is analysed using a standard particle tracking algorithm[20] to obtain distributions of the center-to-center separations of the trapped particles for both experiments. We follow the approach of Grier and coworkers[22] to obtain an autocali-

brated measurement of the colloidal interaction potential by analysing the differences between the distributions in the presence and absence of optically induced forces.

$$\frac{U(r)}{k_{\mathrm{B}}T} = \ln\left[f_0(r)\right] - \ln\left[f_{\mathrm{rl}}(r)\right] \tag{39}$$

where f_{rl} and f_0 are the corresponding distributions of center-to-center separations.
From n measurements of the center-to-center separation r we compute the pair distribution $f_{\mathrm{pair}}(r)$ using the technique of nonparametric density estimation[22,23]:

$$f_{\mathrm{pair}}(r) = \frac{1}{n h_{\mathrm{opt}}} \sum_{i=1}^{n} J\left(\frac{r - r_i}{h_{\mathrm{opt}}}\right) \tag{40}$$

where r_i reflects the separation distance determined from one image i (one measurement); h_{opt} is a smoothing parameter. The estimator's kernel $J[(r - r_i)/h_{\mathrm{opt}}]$ can be any smooth function that satisfies the following conditions: (i) continuous and symmetric around zero (ii) integrable with its maximum J_{max} at zero and (iii) normalized and non-negative[23]. For convenience we choose a Gaussian function of the form:

$$J\left(\frac{r - r_i}{h_{\mathrm{opt}}}\right) = \frac{1}{\sqrt{2\pi}} \exp\left[\frac{(r - r_i)^2}{2h_{\mathrm{opt}}^2}\right] \tag{41}$$

The smoothing parameter h_{opt} reflects the kernel's bandwidth. A proper choice of h_{opt} is crucial. A too large width obscures features in the pair distribution $f_{\mathrm{pair}}(r)$ whereas a too small width yield noisy results. A good trade-off is given by Silverman's rule[23]: $h_{\mathrm{opt}} = \left[4/(3n)\right]^{1/5} \sigma_{\mathrm{r}}$ where σ_{r} is the standard deviation of all separation distances r_i. The benefit of nonparametric density estimation over histograms is (i) the convergence speed; for n data points the statistical error in histograms decreases as $n^{-1/2}$ whereas for nonparametric density estimation the error improves as $n^{-4/5}$ (see refs 22,24). More importantly the nonparametric density estimation does not rely on the choice of discrete bins.

Total interaction potential of the charge stabilized microspheres

Particles suspended in water involve both van der Waals and double layer (dl) electrostatic repulsive interactions which in combination can be described by the well known DLVO theory[25]. The latter is dominantly repulsive for stable suspensions and thus prevents particle coagulation. Equally, in our measurements, this repulsive part dominates at very short distances and in turn this allows us to probe the superimposed light induced attractions, without particles sticking together

irreversibly. For illustration we show in Supplementary Figure 1 a typical DLVO potential for micron sized particles ($2R = 2$ μm) and the superimposed attraction due to random light fields corresponding to the case shown in Fig. 3**a** in the main text. Exact values for the Hamaker constant and the contact potential $U_{\text{dl}}(D = 0)$ are not known and we have chosen reasonable estimates consistent with the observed stability of the melamine particles.

Amorphous turbid layer

The turbid layer at the entry of the light filled cavity is composed of a dense amorphous assembly of PMMA (Polymethylmethacrylate) particles, diameter ~ 0.4 μm. We prepare the sample by filling a hollow rectangle borosilicate glass capillary (CM Scientific) with a height of $\simeq 20$ μm and a width of $w = 200$ μm with a concentrated colloidal suspension with a particle volume fraction of approximately $\phi \approx 0.35$ and then let the suspension dry. We perform scanning electron microscopy on the dry particle layer by breaking the capillary after the experiment. The images (not shown) reveal a densely packed random structure in the bulk of the dried sample and a thin boundary layer with a crystalline structure close to the cell wall. We measure the line-of-sight transmission by collimating the 532 nm laser beam and masking it with a 50 μm pinhole that we place as close as possible (ca. 1 mm) to the front surface of a glass capillary. On the opposite side we record the far field intensity profile by directly placing the sensor of the digital camera. We estimate the line of sight transmission to $T_{\text{los}} = 0.1$ %. We estimate the total transmission by placing a high numerical aperture objective (Nikon 60x PlanApoVC, N.A.= 1.4) on the opposite side of the sample cell in order to maximise the acceptance angle for transmitted light. The collected light is then projected on a screen, imaged and analyzed with the digital camera. From this we obtain an estimate for the diffuse total transmission of $T_{\text{diff}} \sim 1/3$.

Supplementary References

---

[1] Joulain, K. *et al.* Surface electromagnetic waves thermally excited: Radiative heat transfer, coherence properties and casimir forces revisited in the near field. *Surf. Sci. Rep.* **57**, 59–112 (2005).

[2] Setälä, T., Kaivola, M. & Friberg, A. T. Spatial correlations and degree of polarization in homogeneous electromagnetic fields. *Opt. Lett.* **28**, 1069–1071 (2003).

[3] Chaumet, P. C. & Nieto-Vesperinas, M. Coupled dipole method determination of the electromagnetic force on a particle over a flat dielectric substrate. *Phys. Rev. B* **61**, 14119–14127 (2000).

[4] Albaladejo, S. *et al.* Radiative corrections to the polarizability tensor of an electrically small anisotropic dielectric particle. *Opt. Express* **18**, 3556–3567 (2010).

[5] Mishchenko, M. I., Travis, L. D. & Lacis, A. A. *Scattering, Absorption, and Emission of Light by Small Particles* (Cambridge University Press, Cambridge, 2002).

[6] Waterman, P. C. Symmetry, unitarity, and geometry in electromagnetic scattering. *Phys. Rev. D* **3**, 825–839 (1971).

[7] Henkel, C., Joulain, K., Mulet, J.-P. & Greffet, J.-J. Radiation forces on small particles in thermal near fields. *J. Opt. A: Pure Appl. Opt.* **4**, S109–S114 (2002).

[8] Lifshitz, E. M. & Pitaevskii, L. *Statistical Physics Part 2: Landau and Lifshitz Course of Theoretical Physics*, vol. 9 (Pergamon Press, Oxford, 1980).

[9] Kenneth, O. & Klich, I. Opposites attract: A theorem about the casimir force. *Phys. Rev. Lett.* **97**, 160401 (2006).

[10] Emig, T., Graham, N., Jaffe, R. L. & Kardar, M. Casimir forces between arbitrary compact objects. *Phys. Rev. Lett.* **99**, 170403 (2007).

[11] García-Etxarri, A. *et al.* Strong magnetic response of submicron silicon particles in the infrared. *Opt. Express* **19**, 4815–4826 (2011).

[12] Walker, T., Sesko, D. & Wieman, C. Collective behavior of optically trapped neutral atoms. *Phys. Rev. Lett.* **64**, 408–411 (1990).

[13] Sukhov, S., Douglass, K. M. & Dogariu, A. Dipole-dipole interaction in random electromagnetic fields. *Opt. Lett.* **38**, 2385–2387 (2013).

[14] Thirunamachandran, T. Intermolecular interactions in the presence of an intense radiation field. *Mol.*

Phys. **40**, 393–399 (1980).

[15] O'Dell, D., Giovanazzi, S., Kurizki, G. & Akulin, V. M. Bose-einstein condensates with 1/r interatomic attraction: Electromagnetically induced "gravity". *Phys. Rev. Lett.* **84**, 5687–5690 (2000).

[16] Spitzer Jr., L. The dynamics of the interstellar medium. ii. radiation pressure. *Astrophys. J.* **94**, 232–244 (1941).

[17] Hamaker, H. C. The london–van der waals attraction between spherical particles. *Physica* **4**, 1058–1072 (1937).

[18] Ashkin, A. Forces of a single-beam gradient laser trap on a dielectric sphere in the ray optics regime. *Biophys. J.* **61**, 569–582 (1992).

[19] Fällman, E. & Axner, O. Design for fully steerable dual-trap optical tweezers. *App. Opt.* **36**, 2107–2113 (1997).

[20] Crocker, J. C. & Grier, D. G. Methods of digital video microscopy for colloidal studies. *J. Colloid Interface Sci.* **179**, 298–310 (1996).

[21] Biancaniello, P. L. & Crocker, J. C. Line optical tweezers instrument for measuring nanoscale interactions and kinetics. *Rev. Sci. Instrum.* **77**, 113702 (2006).

[22] Polin, M., Roichman, Y. & Grier, D. G. Autocalibrated colloidal interaction measurements with extended optical traps. *Phys. Rev. E* **77**, 051401 (2008).

[23] Silverman, B. W. *Density Estimation for Statistics and Data Analysis* (Chapman & Hall, London, 1986).

[24] Thompson, J. R. & Tapia, R. A. *Nonparametric Function Estimation, Modeling, and Simulation* (Society for Industrial and Applied Mathematics (SIAM), Philadelphia, 1990).

[25] Israelachvili, J. N. *Intermolecular and Surface Forces* (Academic Press, London, 1991).
